# Common causes and types of hand injuries and their pattern of occurrence in Yekatit 12 Hospital, Addis Ababa, Ethiopia

**DOI:** 10.11604/pamj.2019.33.142.18390

**Published:** 2019-06-25

**Authors:** Metasebia Worku Abebe

**Affiliations:** 1 Addis Ababa University, College of Health Sciences, Addis Ababa, Ethiopia

**Keywords:** Hand injury, tendon injury, nerve injury, fractures

## Abstract

**Introduction:**

Hand injuries constitute a major proportion of trauma emergencies in developing countries. The hand establishes the individual in society, allowing them to meet social and economic responsibilities. Previously hand traumas accounted for 12% of major limb traumas in Addis Ababa, Ethiopia; but data on the specific types of tissue injuries and pattern of occurrence of these injuries over the years is limited.

**Methods:**

A retrospective study of sampled 178 patients with hand injury that presented to Yekatit 12 Hospital with hand injuries was done by reviewing the patient's medical records.

**Results:**

Hand trauma is the second commonest injury following burns that present to the Plastic and Reconstructive Surgery unit in Yekatit 12 hospital. It commonly occurs in males with ratio of 4:1. Average age of patients was 24.5 years. The right hand was more commonly injured than the left hand. Home and fall accidents were commonest cause of injury followed by machine injuries. Commonly occurring injuries were tendon injuries followed by fingertip injuries. The number of patients presenting to the hospital with hand injuries has doubled over the two year study period.

**Conclusion:**

The number of hand injury cases that presented to the hospital has doubled over the two years study period. Types of hand injuries presenting to the hospitals ranged from simple lacerations to deep tissue injuries requiring long duration of treatment and rehabilitation which has an impact on the productivity of the younger age group that was identified as the most at risk population.

## Introduction

The hand is a unique fusion of form and function [1]. The hand and wrist are integral components of nearly all human pursuits, including work, leisure, and activities of daily living. This level of involvement makes injuries to these structures common and long-term disability devastating [2]. Injuries of the distal upper extremity range from simple lacerations to complex open blast injuries involving destruction of vital soft tissue nerve and vascular structures [3]. Injuries are the leading cause of death and disability for all age groups except those over age sixty [4]. Injuries are neglected epidemics in developing countries causing more than five million deaths each year, roughly equal to the number of deaths from malaria, HIV/AIDS and tuberculosis combined [5]. Over 90% of these deaths occur in low-and middle-income countries (LMIC), where health systems are least prepared to meet the challenge, and social nets are weak or more often nonexistent [4]. The seminal Global burden of disease and risk factors study estimated that injuries accounted for 15% of all ill heath in the world in 1990 and forecast this to increase to 20% by 2020 [5]. In this era of industrialization and reliance on machines, hand injuries are on the increase worldwide, accounting for 10-15 percent of admissions in emergency departments in the developed countries [6, 7]. These injuries are also among the most common injuries that happen in rapidly developing countries including Ethiopia where hand injuries were demonstrated to contribute to 12% of trauma patients [8, 9]. Injury to the hand leads to loss of function as well as deformity of body image which has a lot of psychological consequences [10]. Road traffic accidents and machinery were found to be the commonest causes of hand injuries in developing countries while leisure and home accidents take precedence in Netherland and Denmark [6, 10, 11]. The hand plays an important role in maintaining body image and sense of identity as well as serving as an organ of communication among other things [9, 10, 12]. Injury to the hand is among the common injuries encountered in the emergency departments of hospitals in the capital city of Ethiopia, Addis Ababa. These injuries mainly affect the young population which is part of the main workforce of the country. Despite this fact, there is limited data on specific types and causes of hand injuries, the pattern of occurrence of the injuries over the years, age groups mainly affected and occupations predisposing patients to hand injuries. Therefore, in the absence of hand injury registry, this study will be conducted to bridge the gaps of data on hand injures with the anticipation of serving as stepping stone to the much needed further studies regarding hand injuries to plan for prevention strategies as development of safety protocols within high and low level industries. It can also be used as a tool to develop proper treatment protocols for those hospitals handling hand injury cases.

## Methods

**Study area and study period:** the study was conducted in Yekatit 12 Hospital Medical College which provides services for patients from both the capital city where it is located and referral cases from other regions of the country. It has nine departments and six units and has 265 beds. Since the year 2000, Plastic, Reconstructive and Hand surgery unit providing burn, cleft, hand and other reconstructive surgery services was established and has started providing emergency hour services by plastic surgery trainees since 2016.

**Study design:** a retrospective study of patients with hand injuries was conducted by reviewing of 178 charts out of the total patients seen with hand injuries over the stated 02 years in Yekatit 12 Hospital. Data was collected from patient's charts seen from September 2015 to August 2017.

**Source and study population:** the source population for the study was patients who have visited Yekatit 12 Hospital emergency department during the study period. The targeted study populations were the patients visiting the hospital for complaints of hand injuries.

**Inclusion criteria:** patients visiting the hospital for complaints of hand injuries.

**Exclusion criteria:** patients presenting with other life threating injuries in addition to the hand injuries.

**Sample size and sampling procedures:** one hundred and seventy eight patients who have visited the hospital with hand injuries and fulfilling the inclusion and exclusion criteria during the study period were included in the study. Sample size was calculated with the assumption that about 12% prevalence of hand injuries with absolute precision of 5%. The needed sample size was estimated to be 162 patients (considering the confidence limits to be 95%). After adding 10% for lost charts and unexpected difficulties, it turned out to be 178. Simple random sampling was employed. Among the patients with hand injures every other patient full filling the inclusion and exclusion criteria was included in the study. If a chart in the sampling frame is missing, incomplete or is excluded; the next patient in the sampling frame was included.

**Data collection procedures:** data was filled out in excel spreadsheet that included all relevant information regarding the demographic data of patients, mechanism of injury, tissues involved, time of presentation, possible risk factors, surgeries performed, total duration of follow up in hospital starting from time of injury types of injuries and mechanism of the injuries. Data was collected with adherence to all ethical prerequisites for the study. Data was collected in Yekatit 12 Hospital Medical College from the sampled 178 charts seen during the timeframe of two years period spanning from September 2015 to August 2017.

**Operational definitions:** hand injuries-injuries to the bone or soft tissue occurring below the wrist joint with exception of tendon injuries outside of this zone. Types of hand injuries-tissues involved in the injury as skin, tendon, nerve, vessel, bone or combination of these injuries. Fingertip injuries-injuries on fingers distal to insertion of the flexor digitorum profondus and extensor tendon.

**Data management and analysis plan:** data was entered in Excel spreadsheet and exported to SPSS (Statistical package for social sciences) version 23 after data cleaning procedures were done. Data was also analyzed using SPSS version 23.

**Ethical consideration:** ethical clearance and approval was obtained from the Institutional review board of Addis Ababa University and Yekatit 12 Hospital Medical College. Anonymity of patients was maintained as they were only identified with their chart numbers during data collection and ethical issues were adhered to during the whole course of conduction of the study.

## Results

Out of the sampled 178 patients with hand injuries 79.8% (142) were male and 20.2% (36) were female with M: F ratio of 4:1. Average age was 24.5 years with minimum age of 1.3 years and maximum age of 75 years. In grouping of the ages 69.1% (123) were between the ages of 18 and 35 while 23.6% (42) were under the age of 18 years and 7.3% (13) were above 40 years of age. 88.8% (158) of participants were from Addis Ababa while 9.6% (17) were from Oromia region while the other 1.6% were from other regions of the country. Hand injuries (284) were the second commonest causes of injuries presenting to the plastic and reconstructive surgery unit in Yekatit 12 Hospital Medical College following burns (535) during the two year study period. The right hand was injured in 51.7% (92) of cases, while left hand was injured in 46.6% (83) patients and 2 patients (1.1%) presented with injury to both hands. 70.2% (125) patients stated home accidents and falls as cause of injury, 17.4% (31) patients sustained the injury while working with a machine and 4.5% (8) sustained the injury in road traffic accidents (Table 1). Of the total 178 participants with hand injury; 30.3% (54) sustained fingertip injury and of these 11.2% (20) had more than one finger with tip injury, 6.7% (12) had index finger tip injury, 5.1% (9) had thumb tip injury, middle and ring with the same rate of occurrence of 3.4% (6) and the little finger tip injury of 1.7% (3). 28.1% (50) had flexor tendon injuries, of which 15% (27) had flexor tendon injury in more than one finger and 3.9% (7) had flexor tendon injury of the index finger.

**Table 1:** Causes of hand injury among patients presenting with hand injury (September 2015 –August 2017)

Cause of injury	Frequency	Percent
Home and fall accident	125	70.2
Assault	12	6.7
Machine	31	17.4
Road traffic accident	8	4.5
Other	2	1.2
Total	178	100

Of the total 28.1% (50) patients with flexor tendon injuries, commonest zones of injuries were Zone II (12.4% (22)) and Zone V (11.2% (20)). 17.4% (31) had extensor tendon injuries of which Zone VI 5.1% (9), IV 3.9% (7) and VII 3.4% (6) were commonest zones injured. Nerve injuries occurred in 19.1% (34) patients, of which 10.1% (18) had digital nerve injury and 5.6% (10) had median nerve injury. 11.8% (21) had injury to a vessel of which 7.9% (14) had digital vessel injury and the index finger vessel injury takes majority with 3.4% (6).29.7% (53) patients had bony injuries (fractures) of which 16.9% (30) had distal phalanx fractures while 5.6% (10) had proximal phalanx fractures, 37.6% (67) of these injuries were combined injuries rather than isolated injuries to a single tissue. Time between injury and presentation to hospital was within 24 hours for 83.1% (148) of patients; of which 71.9% (128) arrived to the hospital within 8 hours and 22.6% (40) arrived within 1 hour of injury while 14.6% (26) presented within 7 days but beyond 24 hours of injury. 70.8% (126) of those patients that needed a surgical procedure received it within 48 hours of the injury but overall mean of duration between presentation and intervention was 4 days. Of the major procedures performed for the patients with hand injuries 25.5% (45) involved tendon repair, 8.9% (16) involved nerve repair and 7.8% (14) involved bony fixation. From the sampled 178 patients; 71.3% (127) occurred in 2009 E.C (September 2016-August 2017) while only 28.6%(51) occurred in 2008 E.C (September 2015-August 2016 G.C) showing a huge increment in the number of hand injuries which is also backed up by total number of patients with hand injuries seen in Yekatit 12 hospital medical college which was doubled from 92 (33.1%) in 2008 EC (September 2015-August 2016 G.C) to 195 (67.9%) in 2009 E.C (September 2016-August 2017).

## Discussion

Hand injuries are the second commonest injuries presenting to plastic and reconstructive surgery unit in the emergency hours at Yekatit 12 Hospital Medical College. Hand injury commonly affects the young working population under the age of 40 years and has a potential of serious handicap due the intricacy of the structures within the hand their complex interaction which is vital for proper function [7, 9, 13, 14]. In this study, hand injuries mainly affected males under the age of 40 years which is consistent with other studies [6-16]. The M:F ratio was 4:1 which is higher than a study in Nigeria [10] but lower than a study in India [14] reflecting variation of culture as well as health seeking behavior. Understanding the environmental etiology is the first step for formulating preventive measures. Commonest cause of hand injury in our study was home and fall accidents (70.2%) which was similar to a study in Poland [7], Denmark and Netherlands [11] and India [14] but in contrast to studies in Uganda [6], Nigeria [10], and Ethiopia [15] which can be explained by the fact that catchment areas of the city referring patients to Yekatit 12 hospital have limited number of industrial sites. Majority (56.7%) of the hand injuries were sharp cuts followed by crush injuries which accounted for 37% of cases which is similar to a study in Poland [7] that identified sharp and crush injuries as commonest mechanisms. Although not the commonest cause of injury, machine injuries contributed to 17.4% of hand injury cases, which is in contrast to a previous study done in Ethiopia [15] that identified machine injuries as the commonest cause. This finding can be explained by the fact that Tikur Anbessa Hospital was the only hospital giving hand surgery services for trauma patients during the period the study was conducted. These findings also indicate that our preventive measures need to emphasize on much needed awareness creation on common causes of hand injury and safety protocols that need to be adhered to both at home and workplaces. The right hand was the more commonly injured hand in this study accounting for 51.7% of the cases which is similar to the finding of studies in Uganda [6], Nigeria [10], India [14].

In this study, 71.9% of patients with hand injury arrived to the hospital within 8 hours of injury and only 22.4% reached the hospital within 1 hour of injury which is better than previous study done in Ethiopia [9] and but much worse than other studies in India and Ethiopia [14, 15]. This demonstrates the necessity of educating the society on early presentation to health facilities, primary health care providers on hand injury cases that need early referral to specialty centers and overall improvement of the referral system of the city. 70.8% of patients that needed a surgical procedure received it within 48 hours of the injury but overall mean of duration between presentation and intervention was 4 days, which is an improvement from a previous study done in Ethiopia [15] but at a worse standing than other studies done in Uganda [6], Poland [7] and Nigeria [10] and is explained by the fact that hand injuries are managed in the same operation theatre with other plastic and reconstructive surgery that have a long waiting list of patients. Since aggressive initial management affords the best chance to optimize final outcome of hand function; this findings highlight the need to reduce the delay between presentation and surgery among our hand injury patients that require surgical intervention either by equipping emergency operation theatre with the necessary surgical equipment or establishing a separate operation theatre for the acute hand injury cases. In our study, various ranges of injuries presented to our hospital, 30.3% sustained fingertip injury which is much higher than study findings in Nigeria [10]. 45.5% of patients had tendon injuries which is much higher than study done in Uganda [6] but lower than the study done in India [14]. Commonest zone of injury on palmar side was Zone II (12.4%) which is contrary to studies done in Uganda and Nigeria [6, 10] which found Zone III to be commonest site of injury. Nerve injuries occurred in 19.1% of the cases in our study which is lower than the study done in India [14] and bony fractures occurred in 29.7% of patients which is lower than studies done in India [14], Ethiopia [15] which can be explained by the fact that commonest cause of injury that was identified in our study was home and fall accidents that are probably low energy injuries and result in soft tissue injuries rather than bone fractures. All these various ranges of injuries require early operative care and prolonged rehabilitation programs indicating the need to strengthen the hand surgery services within the hospital and the country at large; while undertaking preventive measures as safe practices and safety protocols within homes, and, small as well as large scale industries.

## Conclusion

Hand traumas are among the commonly encountered injuries in Yekatit 12 Hospital Medical College-Plastic, Reconstructive and Hand surgery unit. In the absence of hand injury registry, this study aimed to depict the types and causes of hand injuries and the pattern of occurrence which has doubled over the study period. Home and fall accidents followed by machines were found to be commonest causes of hand injury. The types of hand injuries presenting to the hospitals ranged from simple lacerations to deep tissue injuries requiring prolonged periods of treatment and rehabilitation that takes the young population off productivity for a long time.

### What is known about this topic

- Hand injuries are common injuries presenting to emergency units of Hospitals;
- Young males constitute majority of the patients presenting with hand injuries.

### What this study adds

- The commonest types of hand injuries were tendon injuries followed by fingertip injuries;
- The commonest cause of hand injuries were home accidents and falls;
- The number of patients presenting to one of the two hospitals providing hand injury management has doubled over the study period.

## Competing interests

The authors declare no competing interests.
